# The transient intraluminal filament middle cerebral artery occlusion model as a model of endovascular thrombectomy in stroke

**DOI:** 10.1177/0271678X15606722

**Published:** 2015-10-02

**Authors:** Brad A Sutherland, Ain A Neuhaus, Yvonne Couch, Joyce S Balami, Gabriele C DeLuca, Gina Hadley, Scarlett L Harris, Adam N Grey, Alastair M Buchan

**Affiliations:** 1 Acute Stroke Programme, Radcliffe Department of Medicine, University of Oxford, Oxford, UK; 2 Centre for Evidence Based Medicine, University of Oxford, Oxford, UK; 3 Norfolk and Norwich University Teaching Hospital NHS Trust, Norwich, UK; 4 Nuffield Department of Clinical Neurosciences, University of Oxford, Oxford, UK

**Keywords:** Ischemic stroke, middle cerebral artery occlusion, endovascular thrombectomy, cerebral blood flow, animal models

## Abstract

The clinical relevance of the transient intraluminal filament model of middle cerebral artery occlusion (tMCAO) has been questioned due to distinct cerebral blood flow profiles upon reperfusion between tMCAO (abrupt reperfusion) and alteplase treatment (gradual reperfusion), resulting in differing pathophysiologies. Positive results from recent endovascular thrombectomy trials, where the occluding clot is mechanically removed, could revolutionize stroke treatment. The rapid cerebral blood flow restoration in both tMCAO and endovascular thrombectomy provides clinical relevance for this pre-clinical model. Any future clinical trials of neuroprotective agents as adjuncts to endovascular thrombectomy should consider tMCAO as the model of choice to determine pre-clinical efficacy.

## Introduction

Since 1995, recombinant tissue plasminogen activator (rtPA) has been used as a thrombolytic agent to dissolve blood clots that occlude cerebral arteries, leading to reperfusion of the ischemic brain.^1^ However, eligibility for thrombolysis currently stands at approximately 15%, largely due to a lack of efficacy beyond 4.5 h post-stroke, as well as the risk of haemorrhagic transformation.^2^ Other therapies, such as anti-platelet drugs, can be used to prevent further coagulation, but there remains a dearth of novel therapeutic agents. A number of trials have attempted to show neuroprotection of the brain with single drug approaches, including NXY-059 and magnesium sulphate, but no single neuroprotectant has shown any substantial efficacy in a phase III trial. Although intravenous (i.v.) rtPA has the most robust evidence basis for the treatment of acute ischemic stroke, its limitations – including low rates of recanalization – have led to the exploration of other treatments with higher recanalization rates, including endovascular therapy. This approach ranges from intra-arterial thrombolysis^3^ to mechanical thrombectomy using specialized devices that can retract clots and open occluded arteries.^4,5^ Five recently published randomized controlled trials (RCTs) show that endovascular thrombectomy with or without i.v. thrombolysis produces a favourable outcome compared to medical therapy alone.^6–10^ This beneficial effect is correlated with an abrupt recanalization of the occluded artery and subsequent reperfusion of the ischemic brain, which parallels mechanical models of stroke in animals such as the intraluminal filament middle cerebral artery occlusion (MCAO) model. This perspective describes the similarities between the endovascular thrombectomy and transient MCAO (tMCAO) procedures from a cerebral blood flow (CBF) and pathophysiological point-of-view, and provides a rationale for use of filament tMCAO in animals to model the potential outcomes of adjunct therapies to endovascular thrombectomy in humans.

## Endovascular thrombectomy

**Figure 1.**
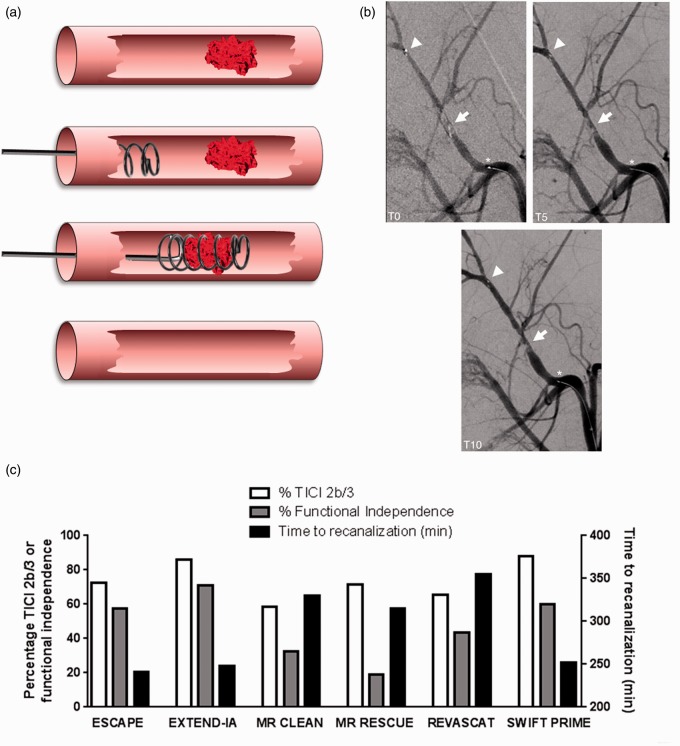
(a) Illustration of endovascular thrombectomy after acute ischemic stroke. Stroke is caused by a clot occluding a major vessel within the CNS. A thrombectomy device (usually helical and surrounded by a network of nitrol filaments) is introduced via a guide cannula in the femoral vein, through the appropriate cerebral vessel (either a vertebral artery or the internal carotid artery) until it reaches the region of the clot. Once the device is advanced so as to be distal to the occlusion, a number of loops are deployed in order to surround the clot. Removal is via the guide cannula, leaving a recanalized vessel. (b) Angiography at 0, 5, and 10 min during stent-retrieval of a thrombus with the Solitaire FR Revascularization Device. There is an immediate restoration of flow at T0 with a progressive increase in recanalization up to T10 (arrow). The arrowhead indicates the distal stent marker distension. The asterisk depicts the location of the proximal stent marker. Reproduced from Mordasini et al.^11^ with permission from *American Journal of Neuroradiology*. (c) Six endovascular thrombectomy trials reported % Thrombolysis in Cerebral Infarction (TICI) 2b/3 scores indicative of recanalization on angiography, % functional independence (modified Rankin Scale 0–2 at 90 days) and time to recanalization. These results reveal that there is a correlation between the success of recanalization and the improvement in functional outcome. A greater chance of recanalization occurred if thrombectomy occurred within 5 h of stroke onset.

With the efficacy of – and eligibility for – rtPA having relatively low rates in clinic, especially for occlusions of larger vessels, the focus over the past decade has been on intra-arterial clot disruption, by either directly infusing rtPA into the artery or using endovascular retrieval devices to mechanically remove the occluding clot from the cerebral circulation. These have been refined over recent years and specialized stent-retriever devices catch and remove the occluding clot (Figure 1(a)), resulting in immediate recanalization of the artery (Figure 1(b))^11^ and reperfusion. Early endovascular thrombectomy trials demonstrated the feasibility and safety of the procedure,^4,5^ but failed to show the benefit of endovascular therapy compared to i.v. thrombolysis alone.^12–14^ Recent overwhelming evidence from five RCTs using stent-retriever devices showed that endovascular thrombectomy for patients with large cerebral vessel occlusion could lead to improved functional independence,^6–10^reduced mortality^8^ and no increase in the rate of symptomatic intracerebral hemorrhage compared to best medical treatment (which could include i.v. rtPA administration). These trials provide unequivocal evidence that the extent of recanalization associates with better clinical outcomes (Figure 1(c)).

In addition to recanalization, MR and CT perfusion imaging used in two trials showed that substantial reperfusion was achieved with endovascular thrombectomy and was associated with improved outcome.^8,10^ Moreover, time to reperfusion is also an important determinant of clinical outcome, with shorter times from stroke onset to initiation of the endovascular procedure associated with a higher proportion of patients with independent functional outcomes. This highlights the importance of timely substantial reperfusion to limit the expansion of the ischemic core following acute ischemic stroke. Given the positive effects of endovascular thrombectomy on ischemic stroke outcome and blood flow, this intervention is likely to become the primary standard treatment alongside i.v. rtPA in centres where it is available.

## In vivo models of stroke

**Figure 2.**
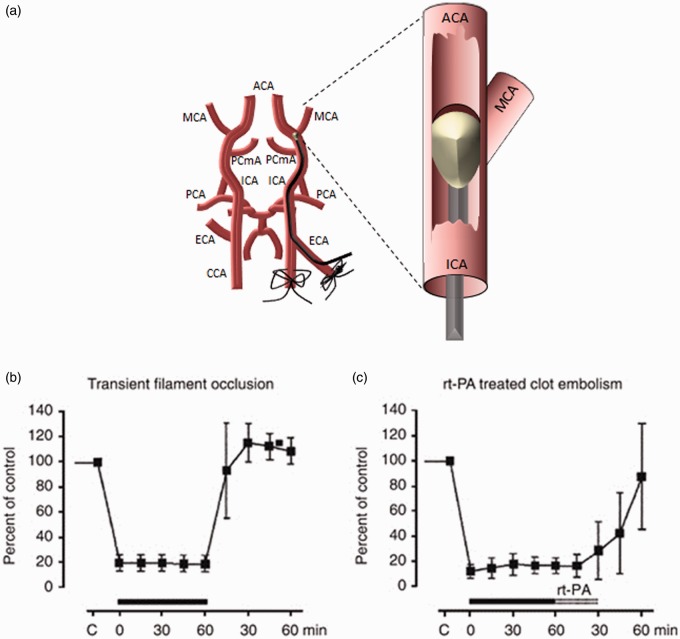
(a) Illustration of the transient middle cerebral artery occlusion model of ischemic stroke in rodents. In the intraluminal filament model, the external carotid artery (ECA) is dissected and cauterized, and the common carotid artery (CCA) temporarily ligated. The internal carotid artery (ICA) is dissected and an arteriotomy is performed in the ECA. The ECA is then reflected so as to run into the ICA and a silicon-tipped nylon filament is introduced into the ICA via the ECA arteriotomy. The filament is advanced until resistance is felt where it occludes the proximal middle cerebral artery (MCA). For transient experiments, the filament remains in place for a set period of time before withdrawal and wound closure. Confirmation of occlusion is usually via concomitant laser Doppler flowmetry. ACA: anterior cerebral artery, PCmA: posterior communicating artery, PCA: pterygopalatine artery. (b) Changes to relative blood flow as a consequence of mechanical MCA occlusion using the transient intraluminal filament model with recanalization achieved by removal of the filament. (c) Changes to relative blood flow as a consequence of clot embolism with recanalization achieved by administration of rtPA. (b) and (c) are reproduced from Hossmann^20^ with permission from Nature Publishing Group.

For more than 50 years, numerous animal models have been used to mimic clinical ischemic stroke, to understand brain pathophysiology and to determine the efficacy of new therapies. The most widely used models produce focal lesions in the parietal cortex and striatum via transient or permanent MCAO, accomplished by mechanical, pharmacological, photothrombotic or embolic means.^15^ MCAO is typically performed in mice or rats, but there are notable examples of primate, canine, ovine and porcine studies using the same methodologies which could have some applicability for translational studies.^16^ In rodents, the intraluminal filament tMCAO model (Figure 2(a))^17^ is the most prevalent, largely because of its non-invasiveness compared to direct surgical occlusion of cerebral vessels, and consistency in lesion size and location compared to embolic models. Despite a vast number of compounds showing therapeutic promise in pre-clinical models,^18^ there has been a notable lack of success, with the exception of i.v. thrombolysis using rtPA, in translating these findings into clinically effective therapies.^19^ There are many putative reasons for these discrepant results, including fundamental inter-species differences, incompatible methods of evaluating the effects of a therapy and, with particular relevance to endovascular thrombectomy, CBF profile differences between transient focal ischemia and recanalization/reperfusion in patients.

The heterogeneity of focal ischemic stroke models is perhaps most prominently reflected in their effects on CBF.^20^ Mechanical occlusion models – utilizing clips or sutures directly placed onto the vessel, or the insertion of an intraluminal filament – produce a characteristic CBF profile (Figure 2(b)), making them some of the more popular models of pre-clinical stroke. At onset, there is a rapid and profound decrease in CBF, maintained for the duration of the occlusion, with perhaps a minimal rise due to collateral vessel dilation. When the vessel is reopened, reperfusion (and in some cases hyperperfusion) occurs almost immediately, followed by a decline back to pre-stroke baseline or to a hypoperfused state. It has been shown that the extent of reperfusion CBF is correlated with a positive outcome following tMCAO,^21^ meaning that differences in reperfused CBF could confound experiments where abrupt recanalization is important. Many studies state that as part of their inclusion criteria, reperfusion CBF must be greater than 70% at onset which provides a more homogeneous group by excluding poorly reperfused animals. Therefore, it is of utmost importance to measure CBF throughout MCAO and subsequent reperfusion so any outcome (behavioural or histological) can be assessed based on the extent of reperfused CBF.

In contrast, embolic and thrombotic models, which have traditionally relied on rtPA to achieve reperfusion, show a very disparate CBF trace: while ischemia is equally rapid, the reperfusion is much more gradual and tends not to display the hyperperfusion seen in mechanical tMCAO (Figure 2(c)). This difference has particular pathomechanistic relevance to reperfusion injury: the rapid influx of oxygenated blood results in the production of superoxide, hydrogen peroxide, reactive nitrogen species and other free radicals, which contribute to neuronal cell death as well as vascular damage in the affected region.^22^ In turn, breakdown of the blood–brain barrier drives further pathological processes, including inflammatory cell recruitment and vasogenic oedema.^23^ Consequently, short-duration mechanical occlusion models feature delayed cell death due to this plethora of secondary damage pathways. By contrast, in embolic models, death occurs primarily during the ischemic period as a direct consequence of anoxia and metabolic failure.^20^ It has therefore been suggested that the root cause of failure of therapeutic strategies in many clinical stroke trials is the reliance of the pre-clinical evidence base on models that fundamentally differ in their mechanism of ischemic damage compared to what is observed in acute stroke patients treated with rtPA.

## Endovascular thrombectomy versus tMCAO

The advent of endovascular thrombectomy as a treatment strategy for acute ischemic stroke provides clinical relevance for mechanical models of stroke. As has been outlined above, CBF changes following endovascular thrombectomy are similar to the CBF changes observed in mechanical MCAO models, such as the intraluminal filament tMCAO model,^17^ where this abrupt occlusion and instant reperfusion are also seen (Figure 2(b)). In comparison with rtPA treatment, where the gradual reperfusion can lead to more severe pathophysiology during ischemia, this rapid restoration in blood flow can lead to reperfusion injury through the rapid influx of systemic immune cells and local free-radical production. It is also thought that haemorrhagic transformation is likely to be the result of rapid reperfusion,^24,25^ however, the recent endovascular thrombectomy trials showed no increase in symptomatic intracerebral haemorrhage compared to best medical treatment (which could include the administration of i.v. rtPA).^6–10^ The positive outcomes of the recent endovascular thrombectomy trials suggest that abrupt restoration of blood flow is more clinically effective than gradual or no reperfusion.^26^ Thus, it seems that the risks of reperfusion injury are outweighed by the benefits of rapid restoration of oxygen and glucose to the ischemic tissue.

Mechanical MCAO models have previously been criticized for not being clinically relevant given the sharp increase in blood flow when the filament or mechanical block has been removed.^20^ However, given the similar CBF and pathophysiological changes between endovascular thrombectomy for human stroke and the rodent intraluminal filament tMCAO model, particularly upon reperfusion, the relevance of the filament model, and in fact any mechanical MCAO model, to clinical practice has become extremely clear. Scepticism regarding the lack of neuroprotection and translational problems with animal models should be reduced with the advent of mechanical thrombectomy as a stroke therapy. The substantial literature describing the ischemic brain and the efficacy of neuroprotective agents following mechanical MCAO and reperfusion, dating back to the 1980s, will now become relevant to clinical practice. The filament tMCAO model is ideal as it is widely validated, shares CBF and pathophysiological profiles with thrombectomy or embolectomy models, and is easy to perform. Moving forward, the intraluminal filament tMCAO model should be considered the gold standard for pre-clinical studies of ischemic stroke treated by endovascular thrombectomy.

## Limitations of pre-clinical models

The limitations of pre-clinical models of stroke, more specifically their lack of translation into effective therapies, have been extensively reviewed elsewhere.^15^ The particular drawbacks of the filament tMCAO model tend to be limited to reproducibility compared to direct occlusion of cerebral vessels. Generally, reproducible lesions, such as those obtained from the permanent proximal electrocoagulation model, produce consistent functional outcomes.^27^ In comparison the tMCAO model outcome is affected by a variety of factors including weight, strain, age and sex of the animal, and filament size. Surgical expertise and finesse also influence the functional outcome as the degree of endothelial damage and precise filament location both affect the severity of injury.^28^ This inconsistency in functional outcome is reflected in the significant variability in lesion size and location^28^ found using the tMCAO model. The variability within the model is further compounded by the vast heterogeneity in technical details between labs. For example, durations of occlusion vary between 30 and 120 min, with longer occlusion times resulting in >20% mortality while brief MCAO produces only selective neuronal death rather than outright pan-necrosis.^29^

In addition, there are certain inescapable differences between patients and rodent models: while the MCA territory is the most commonly affected in ischemic stroke,^30^ the degree of infarction in humans is, on average, 10% of hemispheric volume,^31^ whereas in rodent models it ranges from 10% to 50%.^32^ Similarly, the relative abundance of white matter in humans means that even with proportionally similar lesion volumes, the cytological composition of the affected region is very likely to differ from rodent models. Such differences are problematic; however, our ability to directly assess cerebral pathophysiological changes in patients is largely restricted to post-mortem histological and biochemical analyses, making the use of animal models inevitable.

Fortunately, despite inconsistencies in a given model, some pathological phenomena remain consistent between animal models and the human stroke population. For example, the existence of the ischemic penumbra was originally discovered in rodents^33^ and occurs in both the filament tMCAO model^20,34^ and in human stroke.^35^ There is also a rapid increase in the levels of CNS and systemic cytokines in both stroke patients^36^ and in animal models of stroke,^37^ as well as associated pathological features such as microglial and astroglial activation. These and other similarities underlie the clinical relevance of pre-clinical models and as such the potential power of these tools within stroke research should not be underestimated.

It is important to re-iterate here that the design of pre-clinical stroke experiments will depend entirely on the particular outcome measure desired. If the aim is simply to study the cellular mechanisms of a particular intervention, then single-lab studies or small inter-laboratory collaborations should work. Pre-clinical trials of drugs or interventional studies that aim to continue to phase III must demonstrate global reproducibility across multiple laboratories. The innumerable failures of translation in stroke neuroprotection have led to efforts such as the Stroke Therapy Academic Industry Roundtable (STAIR) criteria,^38^ a list of recommendations for study design and conduct to enhance the reproducibility and quality of individual studies so that findings can be applied to human stroke. However, there is a parallel drive to test compounds in much larger-scale pre-clinical experiments, referred to as ‘phase III pre-clinical trials’. Pre-clinical trials require international collaboration and sharing of tissue and data to minimize the effects of surgical inexperience and variability, and should be placed on the developmental pipeline of an intervention between single-centre pre-clinical studies and multi-centre clinical trials.^19^ The feasibility of international cooperation to conduct multi-centre pre-clinical trials was recently displayed by a European consortium who reported that anti-CD49d therapy was protective in a murine permanent coagulation MCAO model.^39^

## Conclusions

Acute stroke treatment is experiencing a revolution, largely due to the positive results of the five endovascular thrombectomy trials published in early 2015. The therapeutic landscape for stroke patients is changing, and so should the outlook for pre-clinical stroke research. Given the increasing likelihood that endovascular thrombectomy will become a front-line therapy for stroke, alongside i.v. rtPA, further neuroprotection studies and developments in stroke research will still need to use experimental MCAO models. The intraluminal filament tMCAO model is easy to perform and has similar CBF and pathophysiological changes upon recanalization as endovascular thrombectomy in humans. Therefore, any future clinical trials studying neuroprotection as an adjunct therapy to endovascular thrombectomy should consider the filament tMCAO model as the model of choice to determine pre-clinical efficacy.
